# DASS-21 Stress, but Not Anxiety or Depression, Is Associated with Premenstrual Stress

**DOI:** 10.3390/jcm14248619

**Published:** 2025-12-05

**Authors:** Joseph V. Turner, Yolaine Alefsen, Lucas A. McLindon, Damien V. Turner, René Ecochard

**Affiliations:** 1 School of Rural Medicine, University of New England, Armidale, NSW 2351, Australia; 2 Faculty of Medicine, University of Queensland, Brisbane, QLD 4072, Australia; 3 GHU Paris Psychiatrie & Neurosciences, 75014 Paris, France; 4 UFR de Médecine, Université de Paris Cité, 75006 Paris, France; 5 Mater Mothers’ Hospital, Brisbane, QLD 4101, Australia; 6 WA Health, Government of Western Australia, Perth, WA 6027, Australia; 7 CHU de Lyon, 69002 Lyon, France; 8 Laboratoire Biostatistique Santé, Université Claude Bernard Lyon 1, 69100 Lyon, France

**Keywords:** women’s health, distress, stress, depression, anxiety

## Abstract

**Background/Objectives:** Most women experience premenstrual symptoms. We examined how the eleven DSM-5 premenstrual symptoms relate to concurrent indicators of psychological distress. **Methods:** In this retrospective cross-sectional study, 847 participants completed a premenstrual symptom checklist and the DASS-21 between 2011 and 2021. Associations between each DSM-5 premenstrual symptom and DASS-21 depression, anxiety, and stress scores were estimated using univariable and multivariable models, adjusting for the other two DASS-21 subscales and prespecified confounders. **Results:** Eight of the eleven premenstrual symptoms, loss of interest, impaired concentration, depressed mood (*p* < 0.01), loss of control, anxiety, sleep disturbance, emotional lability, and irritability (*p* < 0.05), were independently associated with higher DASS-21 stress scores. After adjusting for stress, no associations were observed with DASS-21 anxiety or DASS-21 depression. **Conclusions:** In this sample, stress was the principal psychological correlate of multiple premenstrual symptoms. These findings indicate co-occurrence rather than causation: the cross-sectional design does not establish directionality, and the observed patterns are compatible with several possibilities (such as stress contributing to, resulting from, or sharing common determinants with premenstrual symptoms). The lack of independent associations with DASS-21 anxiety and depression suggests these symptoms are not merely concurrent expressions of acute anxiety or depressive states. Prospective longitudinal studies are needed to test causal pathways.

## 1. Introduction

Most women of childbearing age experience one or more premenstrual symptoms during their menstrual cycles [1]. More than a hundred different symptoms have been described [2,3,4], and not all women will experience the same symptoms or the same degree of discomfort. Various indicators can be used to differentiate premenstrual disorders: the nature of symptoms (physical, cognitive, affective, behavioral), duration of symptoms (1 to 14 days), number of symptoms, intensity of symptoms, distress associated with the disorder, and the impact of symptoms on daily life [5].

Premenstrual syndrome (PMS) is frequently comorbid with psychiatric conditions such as depressive disorders, anxiety disorders, and stress-related conditions [6,7,8,9,10,11]. However, the precise relationships between symptoms of psychological distress and the manifestation of individual premenstrual symptoms remain unclear. Considering the heterogeneity in premenstrual symptoms, a better understanding of each premenstrual symptom in isolation may contribute to a more individualized understanding of etiological pathways of psychiatric disorders in women experiencing PMS.

The Depression Anxiety Stress Scales-21 (DASS-21) is a validated tool [12] for assessing psychological distress across three dimensions: depression (DASS-21 questions explore the reduction in positive affects, such as loss of pleasure, interest, willpower, enthusiasm, vital drive, self-esteem, enjoyment of life), anxiety (DASS-21 questions explore the physical manifestations of anxiety, such as dry mouth, dyspnea, heart palpitations, tremors, panic sensation, anxious anticipation of a panic attack and fear without an object), and stress (DASS-21 questions explore increased negative affects, such as difficulty in decompressing and relaxing, intolerance of frustration, tendency to overreact, irritability, restlessness, and nervousness).

DASS-21 depression questions are paralleled by premenstrual symptoms such as depressed mood, hopelessness, lethargy, loss of energy, sleep disturbances, impaired concentration, and less interest in usual activities. DASS-21 stress scores are paralleled by premenstrual irritability, feelings of being overwhelmed, mood swings, sleep disorders, and concentration problems, insofar as DASS-21-detected stress corresponds to the nervous tension and emotional dysregulation found in chronic anxiety [13].

Internal consistency for the DASS-21 subscales has been reported in the good to excellent range [14]. Confirmatory factor analyses typically support a three-factor structure corresponding to depression, anxiety, and stress, and cross-cultural research suggests acceptable measurement invariance, albeit with some item-level variation [15].

In this study, we investigated the associations between the eleven premenstrual symptoms outlined in the DSM-5 and the three forms of psychological distress measured by the DASS-21: depression, anxiety, and stress. Our findings aim to provide a deeper understanding of symptoms of PMS and to characterize the psychological distress associated with their manifestations.

## 2. Materials and Methods

### 2.1. Study Design and Setting

For this retrospective cross-sectional study, data from the randomized, therapeutic Pregnancy Achieving Trial (PATrial) were obtained and analyzed. The PATrial was conducted between January 2011 and December 2021 at the Mater Mother’s Hospital in Brisbane, Australia, and approved by the Mater Misericordiae Human Research Ethics Committee (EC00332). Approval for the present study to retrospectively use data from the PATrial was provided by the Mater Misericordiae Human Research Ethics Committee (approval number ERM 72620, amendment AM/MML/72620 V1).

Women entering the PATrial were referred to the Natural Fertility Clinic at the Mater Mother’s Hospital for fertility concerns or recurrent miscarriage. The privacy rights of the participants were observed, and informed consent was obtained for data collection for research purposes. No pilot study was conducted.

### 2.2. Inclusion and Exclusion

The inclusion criteria for the PATrial were adult women, absence of pregnancy after 12 months of trial, or absence of pregnancy of more than 20 weeks after 12 months of trial, or history of 3 or more miscarriages.

The additional inclusion criteria for our study were completion of the DASS-21 and answering the questionnaire exploring the 11 DSM-5 premenstrual symptoms.

The exclusion criteria for the randomized therapeutic trial were subfertility related to a tubal anomaly, subfertility related to the partner, having breastfed in the last 12 months, using contraception, being pregnant, or using fertility-enhancing drugs or supplements.

### 2.3. Factors Studied

#### 2.3.1. Premenstrual Symptoms

The participants in this study were given a self-reported, English questionnaire looking for the presence or absence of the 11 premenstrual symptoms described in the DSM-5 for at least 4 days before the onset of menstruation. These 11 symptoms comprised the validated diagnostic criteria for premenstrual dysphoric disorder. Answers to the self-reported questionnaire were confirmed by a clinician during the following clinical consultation.

#### 2.3.2. DASS-21

The participants in this study were also questioned by a clinician using the DASS-21 questionnaire, concerning their symptoms of stress, depression, and anxiety present over the past 7 days. Each of the twenty-one questions was scored between 0 (“did not apply to me at all”) and 3 (“applied to me very much, or most of the time”), without the woman knowing whether this corresponded to stress, anxiety, or depression. The clinicians then calculated a stress score, depression score, and anxiety score, which corresponded to defined levels of severity [12].

### 2.4. Measures and Covariates

During the initial trial visit, the women were asked to complete the “Women’s medical history questionnaire”, which collected medical, gynecological, and obstetric histories, as well as signs of psychological distress. This included the presence or absence of premenstrual symptoms, the length and regularity of their menstrual cycle, and the presence of symptoms of psychological distress using the DASS-21.

### 2.5. Analytic Strategy

The potential predictive factors, such as age and body mass index (BMI), were classified into categories, for which frequencies were calculated. The pregnancy, childbirth, and miscarriage histories of the participants included in this study were tabulated and presented as percentages.

The number of symptoms in each DASS-21 category presented by the participants was counted and tabulated according to the DASS-21 score classification.

Next, the absence of any significant difference between the DASS-21 responses questioning the premenstrual period and the DASS-21 responses questioning the other periods of the cycle was verified.

The association between the three DASS-21 scores and the presence or absence of premenstrual symptoms was examined by univariate analysis (one-factor logistic regression). Predictive factors for the DASS-21 scores were also examined by univariate analysis.

The covariance structure of the data, that is, the proximity of PMS symptoms to DASS-21 scores, was then examined using factor analysis. This was performed using the ade4 library of the R software (version 4.4.1). Multiple correspondence analysis was performed on the DASS-21 scores. A projection of PMS symptoms was then made as additional columns [16].

Multivariate analysis (logistic regression) was then performed, adjusting the results for the other two DASS-21 scores. In the final step, the multivariate analysis considered the following potential confounding factors: age, parity, past pregnancy terminations, and infertility (defined as trying to conceive for twelve months or more).

Between 2011 and 2021, many serious events occurred in Australia that could have affected the participants’ DASS-21 results and premenstrual stress symptom levels. To protect the analysis against potential bias, we tested how each score changed over time and included the year as a potential confounding factor in the analysis. In undertaking uniformity testing, the inclusion time was divided into three periods: (2010, 2013] (2013, 2016] (2016, 2025], according to terciles. Then, we used a Pearson chi-square test to study the potential evolution of each DASS-21 score. A logistic regression was performed for each PMS symptom separately, with the symptom as the dependent variable and time as the independent variable.

R software (version 4.4.1) was used for all analyses (R Core Team (2024). R: a language and environment for statistical computing. R Foundation for Statistical Computing, Vienna, Austria). The threshold of 0.05 was used to retain differences as statistically significant.

## 3. Results

### 3.1. Participant Flow

Between January 2011 and December 2021, 2666 women received the Women’s medical history questionnaire, of which 847 (32%) met the inclusion criteria.

### 3.2. Description of the Population

Over 80% of the 847 women were aged between 25 and 40. Almost half of them had a normal BMI, a quarter were overweight, and almost a quarter were obese. Almost 80% of the women had already been pregnant, but less than half of the 847 women had already given birth. More than half had experienced at least one miscarriage, and 17% had undergone an abortion. More than half of them were classified as being infertile since they had been trying to conceive for over a year.

### 3.3. Premenstrual Symptoms

Of the 847 women who completed both questionnaires, 89% had at least one premenstrual symptom.

The two most frequent symptoms were physical symptoms and irritability, both present in over half the women, followed by fatigue, emotional lability, and cravings, each present in around one-third of the women. Infrequent symptoms, present in less than 6% of the women, included feeling a loss of control/overwhelmed, less interest in usual activities, and difficulty with concentration.

### 3.4. Symptoms of Stress, Anxiety, and Depression (DASS-21)

More than 75% of the women had normal DASS-21 scores for each category. Considering DASS-21 scores in the abnormal range, of the 847 women, 20% had symptoms of depression, 25% had symptoms of anxiety, and 20% had symptoms of stress (Table 1).

The majority had mild to moderate symptoms (15% for depression, 20% for anxiety, and 18% for stress). Few had severe or extreme symptoms (less than 6% for anxiety and depression, and less than 2% for stress).

### 3.5. Univariate Analysis of Predictors of DASS-21 Score Results

Table 2 shows that there was no significant association between population characteristics and DASS-21 scores, except for older age, which was correlated with a slightly lower DASS-21 score, and the presence of at least one premenstrual symptom, which was correlated with a slightly higher DASS-anxiety score.

### 3.6. Univariate Relationship Between Each Premenstrual Symptom and DASS-21 Scores

**Figure 1:**
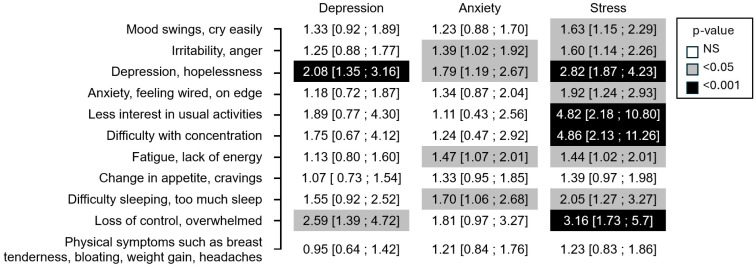
Result of the DASS questionnaire (mild, moderate, or severe vs. normal) and occurrence of PMS symptoms odds ratio [95% confidence interval] (*N* = 847)—univariate analysis.

There was a significant correlation between certain premenstrual symptoms and DASS-21 scores (Figure 1):

- Premenstrual depression/hopelessness was correlated with all three DASS-21 scores, stress, depression, and anxiety;
- The feeling of loss of control/being overwhelmed in the premenstrual phase was correlated with the DASS-21 stress and DASS-21 depression scores;
- Irritability, fatigue, and sleep disorders correlated with the DASS-21 stress and DASS-21 anxiety scores;
- Impaired concentration, loss of interest in usual activities, and anxiety were correlated only with the DASS-21 stress score.

All symptoms, except physical symptoms and a change in appetite/cravings, were significantly correlated with the DASS-21 stress score.

The first axis, obtained using a factor analysis to examine the covariance structure of the three dimensions of the DASS-21 scores, contrasts the positivity of the three scores with their common absence. The second axis contrasts stress and depression with anxiety.

Projection of PMS symptoms onto these coordinates revealed a relationship between some PMS symptoms and stress and depression, mainly as follows: impaired concentration, loss of interest in usual activities, the feeling of loss of control/being overwhelmed, and depression/hopelessness.

### 3.7. Multivariate Relationship Between Each Premenstrual Symptom and DASS-21 Scores

**Figure 2:**
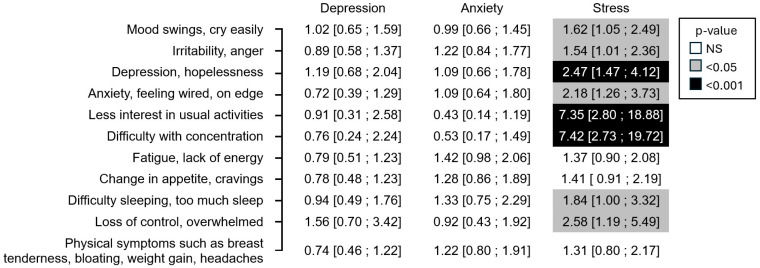
Result of the DASS questionnaire (mild, moderate, or severe vs. normal) and occurrence of PMS symptoms odds ratio [95% confidence interval] (*N* = 847)—multivariate analysis.

When the multivariate analysis adjusted for the other two DASS-21 scores (Figure 2), only the association between the DASS-21 stress score and certain premenstrual symptoms remained significant. Thus, eight premenstrual symptoms were significantly more present in women with stress than in women without stress. The three symptoms most correlated (i.e., higher odds ratio) with the DASS-stress score were difficulty with concentration, less interest in usual activities, and depressed mood with hopelessness (*p* < 0.001). These were followed by loss of control/feelings of being overwhelmed, anxiety/feeling wired on edge, sleep disturbance, mood swings, and irritability/anger (*p* < 0.05).

### 3.8. Multivariate Analysis Accounting for Each Predictor of DASS-21 Outcome

**Figure 3:**
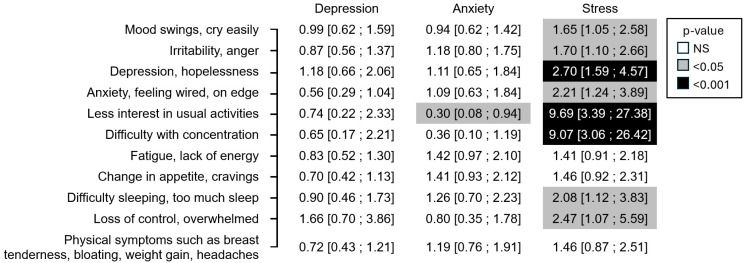
Result of the DASS questionnaire (mild, moderate, or severe vs. normal) and occurrence of PMS symptoms odds ratio [95% confidence interval] (*N* = 847)—multivariate analysis adjusted for confounding factors.

After adjusting the previous multivariate analysis for confounding factors, the results changed very little, as shown in Figure 3. Less interest in usual activities became more correlated with stress than difficulty with concentration and became inversely correlated with anxiety. The results remained almost unchanged when the year was added as an additional confounding factor.

### 3.9. Uniformity Testing

The *p*-values for uniformity testing were 0.1284, 0.6055, and 0.522, respectively, for the depression, anxiety, and stress scores. Logistic regression for each PMS symptom against time did not reveal any statistically significant association (Table 3).

## 4. Discussion

This study demonstrated that amongst a population of women seeking assistance for subfertility and miscarriage, there was a significant association between stress, as measured by the DASS-21, and premenstrual symptoms, except for physical symptoms, appetite disturbance, and lethargy/lack of energy. In addition, after adjusting for stress, we observed no association between premenstrual symptoms and DASS-21 anxiety, nor between premenstrual symptoms and DASS-21 depression.

### 4.1. The Link Between Premenstrual Symptoms and DASS-Stress

Some of the DASS-21 items are common to the 11 premenstrual symptoms examined. Irritability, feeling out of control/overwhelmed, and anxiety are premenstrual symptoms that are also explored through DASS-21 stress. These are symptoms for which our study showed a statistical link with DASS-stress, in both univariate and multivariate analyses.

The results of this study show that DASS-21 stress is also associated with other non-common premenstrual symptoms, and primarily with impaired concentration, lack of interest in usual activities, depressed mood, and then mood swings and sleep disorders, which is surprising since these are not symptoms detected by the DASS-21 stress items. Thus, suffering from impaired concentration, lack of interest and pleasure in usual activities, and depressed mood during the premenstrual period would be associated in some women with DASS-21 stress symptoms also present outside the premenstrual phase. Clinically, DASS-21 stress encompasses nonspecific symptoms of general distress, indicative of a state of chronic anxiety and emotional dysregulation characterized by difficulty in relaxing, a tendency to overreact, intolerance to frustration, heightened susceptibility, and a persistent feeling of being on edge.

To explain these findings, we propose three postulations: (1) premenstrual symptoms may result from or be exacerbated by stress; (2) stress could be the consequence of premenstrual symptoms; and (3) both stress and premenstrual symptoms may arise from common underlying factors.

#### 4.1.1. Postulation 1: Premenstrual Symptoms Can Be a Result of, or Made Worse by, Stress

Stress activates the hypothalamic–pituitary–adrenal (HPA) axis, leading to the production of cortisol. Chronic stress-induced dysregulation of the HPA axis can affect the gonadotropic axis, thereby impacting ovulation and progesterone production [17]. Progesterone is the precursor of allopregnanolone, which is a neuroactive steroid known to be implicated in premenstrual symptoms [18]. Repeated and chronic stress is known to lead to a significant reduction in serum concentrations of allopregnanolone. Moreover, allopregnanolone concentration is reduced in women with PMS [19].

The stress detected by the DASS-21 is manifested as difficulty in relaxing, a tendency to overreact, intolerance to frustration, susceptibility, agitation, and nervousness. One can imagine that living in this state of chronic stress and emotional hyper-reactivity can impact mood, concentration, and interest in usual activities, which could be exacerbated if gonadal hormonal fluctuations are impacted by dysregulation of the hypothalamic–pituitary–adrenal axis.

This postulation would be consistent with several studies in the literature. A longitudinal study showed a temporal relationship between perceived stress and perimenstrual symptoms in the next cycle [6]. Other studies explored the impact of various psychological and cognitive factors that promote stress and the onset of premenstrual symptoms. These factors include emotional dysregulation [20], tendency to ruminate [21], neuroticism [22,23,24], and external locus of control [9]. Neuroticism is a personality trait that can be defined as an individual’s inadequate response to stress, which can result in feelings of instability and emotional insecurity, high levels of anxiety, an increased tendency to experience anger, as well as psychosomatic symptoms [23]. The link between trauma and abuse and PMS has also been studied. One recent study suggested a strong association between childhood trauma and abuse and premenstrual dysphoric disorder (PMDD) [25], corroborating previous studies that had similar findings [26,27]. It has been found that early life trauma can alter the hypothalamic–pituitary–adrenal (HPA) axis, which has been implicated in both stress and reactivity [28]. For women who had experienced childhood sexual abuse, dysregulation of oxytocin secretion from the pituitary gland has been linked with the severity of premenstrual symptoms [29]. A study showed that women suffering from PMS tend to perceive their family and work environments as more stressful. At home, women suffering from PMS describe more rigid, moralistic, and stressful family functioning than women without PMS. In terms of work, women suffering from PMS describe more pressure at work than women without PMS, with less room for autonomy and innovation [30]. Couples whose wives suffer from PMS are less satisfied overall with the quality of their relationship than couples without PMS, irrespective of the phase of the cycle [31].

Nevertheless, this different perception of the environment could also be intertwined with some of the psychological factors mentioned above, which could influence these women’s negative perception of their environment [32].

#### 4.1.2. Postulation 2: Stress Could Be the Consequence of Premenstrual Symptoms

Severe PMS could have an impact on psychological wellbeing in other phases of the cycle. There is considerable literature exploring the impact of PMS on quality of life, with negative repercussions on work performance, social functioning, and family and conjugal life [31,33,34]. Considering the three premenstrual symptoms most strongly associated with DASS-21 stress (depressed mood, lack of interest in usual activities, and impaired concentration), we can understand why they could be a source of stress that may persist the rest of the cycle. Experiencing these three premenstrual symptoms for between 4 and 14 days of a cycle could consistently impair work performance and impact a woman’s professional and hierarchical relationships, her integration and recognition within a team, and thus, her self-confidence. In addition, the daily management of family and home life often falls disproportionately on women, which can represent a significant psychosocial burden [35]. In the case of impaired concentration, loss of interest in usual activities, and depressed mood during the premenstrual period, this daily burden can be felt as heavier and a source of stress that may persist for the rest of the cycle in the form of difficulty relaxing and emotional hyper-reactivity. In terms of marital and intimate life, several studies have looked at the link between PMS and the quality of a couple’s relationship. A 2018 study [31] showed a link between PMS and the quality of a couple’s relationship. This manifests itself differently in men and women. In women, a decline in the quality of a couple’s relationship is perceived during the symptomatic premenstrual week (less satisfaction, less sharing of good experiences, and more conflict). When comparing women with and without PMS, there was a significant difference between the two groups, with less overall satisfaction over the whole cycle in women with PMS (but no difference in sharing of good experiences or frequency of arguments). Men did not report any deterioration in relationship quality during the premenstrual period compared with the rest of the cycle. However, men whose wives suffer from PMS report a poorer quality of couple relationship more globally (less satisfaction and less sharing of good experiences), over the whole cycle, compared with men whose wives do not suffer from PMS. Overall, this study shows that only women perceive a significant difference in their couple relationship between the premenstrual period and the rest of the cycle, but that the level of satisfaction perceived by both men and women in the couple is lower overall in couples whose wife suffers from PMS than in couples without PMS. These results do not, however, allow us to conclude whether it is the premenstrual symptoms that reflect negatively on the couple’s overall relationship, or whether it is the emotions linked to a globally unsatisfactory couple relationship and the resulting stress that are then exacerbated during the period of premenstrual vulnerability.

The results of our study may provide some clues. Based on our study, in support of this postulation, a depressed mood, associated with sadness, hopelessness, and a negative view of the world, may modify women’s perception of their couple relationship, focusing more easily on difficulties at this time of the cycle. A “loss of interest in usual activities”, which clinically corresponds to anhedonia, could explain the reduced frequency of sharing good times as a couple, and it may also be linked to a reduced libido. This echoes the results of a study that showed lower sexual satisfaction in women suffering from PMS [36]. Understandably, this can have an impact on the couple’s overall satisfaction and can therefore be a stress factor. Furthermore, if the period of PMS has generated conflict within the couple through increased irritability (one of the most frequent symptoms), we can imagine that the interpersonal bond may be strained, affecting the quality of the relationship and being a source of stress more generally.

#### 4.1.3. Postulation 3: Stress and Premenstrual Symptoms Could Result from Common Factors

In this postulation, common factors would generate both stress and PMS, based on common pathophysiological mechanisms.

One possible mechanism could be that of genetic susceptibility, for example, single nucleotide polymorphisms (SNPs) in estrogen receptor alpha (ERα) [37], and the 5-HTTLPR5 gene (promoter of the serotonin transporter) [19], which have been identified by various authors as being correlated with PMS and certain personality traits, such as neuroticism.

Another potential explanation is the interaction between the hypothalamic–pituitary–gonadal axis and the serotonergic and gamma-aminobutyric acid (GABA)–ergic system [18]. Decreased levels of allopregnanolone, resulting in reduced GABA receptor activity and inhibition of the inflammatory response to acute stress [38], may correspond to evidence that some premenstrual symptoms can be explained by reduced sensitivity of GABA receptors to allopregnanolone [39].

Several studies have demonstrated functional variability in the amygdala’s response to stress in women with PMDD vs. healthy controls [40].

Notably, a recent study investigating the relationship between PMS and hormonal levels revealed that the five premenstrual symptoms most strongly associated with DASS-21 stress are also correlated with a lower progesterone/estrogen ratio seven days after ovulation [41].

### 4.2. The Absence of a Link Between Premenstrual Symptoms and DASS-Anxiety

Two premenstrual symptoms are also explored through the DASS-21 anxiety questionnaire: anxiety/tension/feeling on edge and feeling overwhelmed/out of control. However, no correlation was found between these premenstrual symptoms and their expression on the DASS-21 anxiety after adjustment for stress.

Other premenstrual symptoms are associated with the DASS-21 anxiety score in univariate analysis, but there is no longer a significant association in multivariate analysis.

The DASS-21 anxiety score in this study addresses the acute aspect of anxiety, i.e., the physiological hyperexcitability that manifests itself during acute anxiety, with symptoms akin to anxiety attacks and panic attacks. The findings suggest that premenstrual symptoms are not caused by a predisposition to acute anxiety, do not cause acute anxiety, and are a distinct entity from the processes of anxiety attacks and acute anxiety. These results contrast with other studies in the literature, which report on correlations between panic disorder and PMDD [42]. However, these literature studies differed from our study in that they utilized a smaller sample size and did not adjust for stress.

### 4.3. The Absence of a Link Between Premenstrual Symptoms and DASS-Depression

Premenstrual symptoms, such as decreased interest in usual activities, difficulty concentrating, and a depressed mood, are all indicative of depression. Four questions in the DASS-21 depression questionnaire assess depressed mood, while three questions focus on decreased interest in usual activities. However, our results do not indicate any correlation between these three symptoms and DASS-21 depression scores. Thus, it seems unlikely that these premenstrual symptoms are an exacerbation of depressive feelings.

Nonetheless, since these symptoms are strongly correlated with DASS-stress scores, it is questionable whether they may be the cause or consequence of stress, or if there could be common mechanisms leading to both stress and these symptoms. Conversely, our results did not reveal any correlation between premenstrual fatigue, cravings, and physical symptoms and DASS-21 scores. Therefore, these symptoms appear to be independent of psychological distress.

Two premenstrual symptoms (depressed mood/hopelessness and less interest in usual activities) are explored by DASS-depression. In the univariate analysis, only depressed mood was correlated with DASS-depression, but not loss of interest in usual activities. In the multivariate analysis, no statistical relationship was found between DASS-depression and premenstrual symptoms. These results contrast with other studies in the literature that report correlations between PMDD and depression [43] or find longitudinal correlations between self-reported PMS and depression later in life [7]. It is also worth remembering that PMS is associated with an increased risk of postpartum depression [44]. However, these literature studies differed from our study in that they did not adjust for stress.

The DASS-21 depression score examines the reduction in positive affects found in depression, with sadness, loss of vital drive, anhedonia and abulia, and a negative view of oneself, the world, and the future. The absence of a statistical link might suggest (1) that premenstrual symptoms are not the result of a lack of positive affect due to depression, (2) premenstrual symptoms do not cause a decrease in positive affect for the remainder of the cycle, and (3) premenstrual symptoms are a distinct entity.

### 4.4. Strengths and Limits of This Study

Compared with the existing literature on the subject, this is the largest cohort of women to our knowledge. This study is novel in that it assesses the associations between each of the 11 premenstrual symptoms of the DSM-5 and the DASS-21. Indeed, previous studies that have explored the links between PMS and DASS have done so either by observing the absence or presence of PMS [9], by exploring the severity of PMS [18], or by exploring three main categories of symptoms [45]. Individual assessment of each of the 11 symptoms of PMS has not previously been undertaken. The clinical diagnosis of PMS is blunt, since it is defined by a collection of symptoms that may not be underpinned by the same pathophysiological mechanisms.

One limitation was that data on premenstrual symptoms were collected at a single point, whereas clinical recommendations indicate that the diagnosis of PMS should be validated by prospective assessment tools over at least two months [46]. In addition, it is possible that DASS-21 scores collected outside the premenstrual phase may vary from scores collected during the premenstrual phase. Furthermore, it is noted that DASS-21 scores relate to the week preceding administration of the questionnaire, and data relating to psychological distress outside of this time, as well as symptoms of chronic psychological distress, were not available in the current study. Over such a long period of patient inclusion, both PMS and DASS-21 scores may change, resulting in an artefactual correlation. However, a careful check using the Pearson chi-square test has not found any such evolution. This limits the risk of inherent bias due to the long period of data collection. Care should also be taken in generalizing these results outside the population of women presenting with fertility or miscarriage concerns. Since ethnic identification, cultural background, and socioeconomic measures were not collected, these also limit the generalizability of results.

The type of analysis described in the current study reveals two types of collinearity: vertical collinearity (among DASS scores, in our case) and lateral collinearity (between DASS scores and PMS symptoms) [47]. If all PMS and DASS scores were considered together in a structural equation modeling (SEM) model, it would be necessary to test for multicollinearity. This is important because PMS symptoms are part of the DASS measurement, meaning that collinearity occurs at a semantic level. This was one of the reasons these symptoms were initially studied separately. In a second step, the symptoms were introduced together, and their interaction was then tested in a logistic regression model. In the future, a careful SEM model could be employed to examine the relationship between PMS and DASS scores more broadly. Before conducting such an analysis, careful consideration of vertical and lateral collinearity would be necessary.

Finally, an important limitation remains, regardless of the efforts made to protect against potential bias: the symptoms of premenstrual stress that were tested are in fact symptoms of stress and general distress and are not specific to premenstrual stress.

## 5. Conclusions

The current study showed a statistically significant association between psychological distress and premenstrual symptoms. More specifically, most of the premenstrual symptoms retained as DSM-5 criteria (except fatigue, craving, and somatic symptoms) are associated with stress, as measured by the DASS-21 stress score, which reflects a state of tension, emotional dysregulation, and negative affect present in both depression and anxiety. This study does not allow us to determine whether it is psychological distress that contributes to the development of these symptoms or whether it is these symptoms that are the source of psychological distress. There are reasonable arguments to suggest that common pathophysiological mechanisms are at work and that stress is possibly both a cause and a consequence of premenstrual symptoms. The findings presented here must be interpreted in light of the retrospective cross-sectional nature of the dataset, demonstrating concurrent relationships within the data rather than causal relationships.

Nevertheless, in clinical practice, this highlights the importance of assessing the presence of premenstrual symptoms in patients suffering from chronic stress and affective disorders. Conversely, it also shows the importance of looking at mental health, psychosocial stressors, history of abuse and trauma, and assessing the psychological distress of women who may come to consult for premenstrual symptoms.

Further research would be beneficial to our understanding of the heterogeneity in premenstrual syndromes, to see, for example, which symptoms are most frequently associated with each other, to assess the link between psychological distress and the number of associated symptoms, and to ascertain whether certain symptom associations are more closely linked than others with psychological distress. Assessing the intensity of symptoms, as well as their duration, may also help deepen our understanding and, ultimately, management of PMS.

## Figures and Tables

**Table 1:** DASS-21 response categories (*N* = 847).

	Categories	*N*	%
Depression score	0–4: Normal	683	(80.6)
	5–6: Mild	70	(8.3)
	7–10: Moderate	58	(6.8)
	≥11: Severe/extreme	36	(4.3)
Anxiety score	0–3: Normal	635	(75)
	4–5: Mild	102	(12)
	6–7: Moderate	63	(7.4)
	≥8: Severe/extreme	47	(5.5)
Stress score	0–7: Normal	673	(79.5)
	8–9: Mild	66	(7.8)
	10–12: Moderate	92	(10.9)
	≥13: Severe/extreme	16	(1.9)

**Table 2:** Predictors of higher depression, anxiety, or stress scores—univariate analysis.

		Depression Score		Anxiety Score		Stress Score	
Characteristic		Correlation	*p*-Value	Correlation	*p*-Value	Correlation	*p*-Value
Age		−0.12 (−0.19;−0.05)	0.0005 *	−0.11 (−0.17; −0.04)	0.0018 *	−0.07 (−0.14; −0.00)	0.0408 *
	Class	Mean (5; 95% CI)	*p*-value	Mean (5; 95% CI)	*p*-value	Mean (5; 95% CI)	*p*-value
At least one PMS symptom	no	2.45 (1.76;3.15)	0.807	1.85 (1.24;2.46)	0.022 *	4.30 (3.47;5.12)	0.250
	yes	2.54 (2.30;2.78)		2.60 (2.39;2.81)		4.81 (4.52;5.09)	
BMI categories	Normal weight (18.5–24.9 kg/m^2^)	2.51 (2.17;2.84)	0.325	2.51 (2.22;2.80)	0.755	4.81 (4.42;5.20)	0.834
	Obese (>30 kg/m^2^)	2.32 (1.84;2.841)		2.31 (1.89;2.74)		4.69 (4.12;5.26)	
	Overweight (25–29.9 kg/m^2^)	2.77 (2.32;3.22)		2.59 (2.20;2.99)		4.75 (4.21;5.28)	
	Underweight (<18.5 kg/m^2^)	1.5 (−0.06;3.06)		2.833 (1.47;4.19)		3.94 (2.10;5.79)	
Gravidity	0	2.58 (2.09;3.08)	0.813	2.17 (1.73;2.60)	0.086	4.60 (4.01;5.18)	0.57
	1	2.39 (1.90;2.88)		2.36 (1.93;2.79)		4.57 (3.99;5.15)	
	2 or more	2.57 (2.27;2.87)		2.71 (2.44;2.97)		4.88 (4.52;5.24)	
Parity	0	2.72 (2.43;3.02)	0.154	2.56 (2.29;2.82)	0.914	4.79 (4.44;5.15)	0.928
	1	2.25 (1.84;2.67)		2.46 (2.10;2.83)		4.68 (4.18;5.17)	
	2 or more	2.32 (1.66;2.98)		2.49 (1.91;3.07)		4.75 (3.97;5.54)	
Number of miscarriages	0	2.64 (2.30;2.97)	0.360	2.46 (2.17;2.76)	0.707	4.72 (4.33;5.12)	0.671
	1	2.68 (2.17;3.20)		2.69 (2.24;3.14)		4.99 (4.39;5.60)	
	2 or more	2.30 (1.92;2.69)		2.50 (2.15;2.84)		4.66 (4.19;5.12)	
Trying to conceive for ≥ 12 months	no	2.59 (2.24;2.93)	0.680	2.74 (2.43;3.04)	0.062	4.98 (4.57;5.39)	0.149
	yes	2.49 (2.19;2.79)		2.35 (2.09;2.62)		4.58 (4.22;4.94)	
Ectopic pregnancy	no	2.50 (2.27;2.74)	0.249	2.51 (2.30;2.71)	0.675	4.75 (4.47;5.02)	0.743
	yes	3.17 (2.06;4.27)		2.72 (1.75;3.69)		4.97 (3.66;6.28)	
Abortion	0	2.44 (2.19;2.69)	0.121	2.47 (2.25;2.69)	0.332	4.67 (4.37;4.96)	0.062
	1	2.82 (2.20;3.43)		2.67 (2.12;3.21)		4.82 (4.08;5.55)	
	2 or more	3.59 (2.36;4.81)		3.24 (2.16;4.32)		6.45 (4.99;7.90)	

* statistically significant, <0.05.

**Table 3:** Logistic regression for each PMS symptom against time.

PMS Symptom	*p*-Value
Mood swings, cry easily	0.885
Irritability, anger	0.094
Depression, hopelessness	0.765
Anxiety, feeling wired on edge	0.546
Less interest in usual activities	0.273
Difficulty with concentration	0.223
Fatigue, lack of energy	0.186
Change in appetite, cravings	0.809
Difficulty sleeping, too much sleep	0.199
Loss of control, overwhelmed	0.280
Physical symptoms such as breast tenderness, bloating, weight gain, headache	0.076

## Data Availability

This study does not have ethical approval to share the de-identified data publicly. It is possible that other researchers may access the data, but this must occur by contacting the research team/ethics committee and providing appropriate justification.

## Abbreviations

The following abbreviations are used in this manuscript:

DASS-21	Depression Anxiety Stress Scales-21
PMS	premenstrual syndrome
BMI	body mass index
PMDD	premenstrual dysphoric disorder
HPA	hypothalamic–pituitary–adrenal
DSM-5	diagnostic and statistical manual, fifth edition
